# Probe into the treatment of tinnitus by acupuncture combined with medicine under the mechanism of pathophysiology: A review

**DOI:** 10.1097/MD.0000000000039832

**Published:** 2024-09-27

**Authors:** Wenhui Hu, Dongye Xu, Qingchang Xing

**Affiliations:** a The Eighth Medical Center of Chinese PLA General Hospital & Medical School, Beijing, China.

**Keywords:** acupuncture, comprehensive treatment, pathophysiology, tinnitus, traditional Chinese medicine

## Abstract

Tinnitus is a common medical disorder. The risk factors include hearing loss, ototoxic medications, head injuries, and depression. Therefore, ear disorders, anxiety, and depression should be considered in the treatment of tinnitus. Although considerable research has been conducted on the pathogenesis and treatment of tinnitus, there is currently no effective treatment. Traditional Chinese medicine (TCM) has a certain effect on tinnitus and a large number of clinical trials have been conducted. Its treatment methods vary and include TCM, acupuncture, and music therapy. TCM treatment of tinnitus usually takes the method of comprehensive treatment, not only relying on drugs but also safety. Therefore, this review explores the treatment of tinnitus using acupuncture combined with medicine, based on the new pathophysiological mechanism of tinnitus.

## 1. Introduction

Tinnitus is the perception of sound, in the head or ears, from no source other than body. The 2 basic types of tinnitus are primary and secondary.^[1]^ The sensation of primary tinnitus is entirely subjective, originating somewhere in the auditory system and is confined to the auditory pathway. The American Academy of Otolaryngology and Head and Neck Surgery Foundation defines primary tinnitus as idiopathic, which may be related to sensorineural hearing loss. Or it may not.^[2]^ Secondary tinnitus involves an underlying mechanical source within the head or neck that transmits an acoustic signal through bone conduction to the auditory end organ where it is detected and processed, similar to any external sound.

The word “tinnitus” originates from the Latin verb tinnire, which describes the conscious perception of hearing in the absence of a corresponding external stimulus. Tinnitus can be subjective, when the experience is personal, or less commonly objective, when the observer can hear the tinnitus. This feeling is usually of a basic nature, although, in some cases, more complex sounds, such as music, are perceived.^[3]^ Tinnitus can sometimes be a rhythmic or pulsing sound. Pulsatile tinnitus^[3]^ can be synchronized with the heartbeat, in this case, myoclonus of the middle ear or palatal muscles.^[4]^ Tinnitus can be persistent or intermittent, and many sufferers experience more than 1 sound. These sounds can be located in 1 or both ears or in the center of the head.

With the continuous progress in scientific research, the understanding of neuroimaging and the pathophysiology of tinnitus has deepened. However, the mechanism of tinnitus morbidity has not yet been fully elucidated. Based on the new pathophysiological mechanism of modern medicine, this study explored the treatment of tinnitus by acupuncture combined with medicine.

## 2. Epidemiology of tinnitus

To date, there has been no authoritative large-sample epidemiological study on tinnitus. An overview of the results of epidemiological studies on tinnitus is limited to population-specific studies. Hoffman and Reed^[5]^ investigated epidemiological studies related to tinnitus. Their investigation is relatively comprehensive. Their major findings include the following: (1) factors highly associated with the presence and severity of tinnitus include the degree of hearing loss, exposure to high levels of occupational and nonoccupational noise, and general health; (2) other factors associated with tinnitus include cardiovascular and cerebrovascular disease, medication, ear infection and inflammation, hyperthyroidism and hypothyroidism in the head or neck, Meniere disease, otosclerosis, sudden deafness, and vestibular Schwannoma; (3) genetic factors may be associated with tinnitus; (4) age may not be associated with tinnitus once hearing loss is taken into account; (5) the prevalence of chronic tinnitus in the adult population is 10% to 15%; and (6) veterans are at a significantly higher risk of chronic tinnitus.

McCormack et al^[6]^ conducted a systematic review of all studies that reported the incidence of tinnitus in adults from 1980 to 2015. Tinnitus was reported in 5% to 43% of all studies. Only 12 of these studies used a consistent definition of tinnitus, with an incidence rate between 12% and 30%. In addition, there were 8 different definitions of tinnitus: 34% of the studies defined tinnitus as “lasting more than 5 minutes at a time..” Researchers have noted that tinnitus is widely defined and inconsistently reported. In addition, the heterogeneity of the data prevented pooling of data across studies for meta-analysis. These studies used different tinnitus diagnostic criteria, considered different age groups, and analyzed and reported them differently. Therefore, it was not possible to estimate the global incidence of tinnitus in this study.

Currently, the largest and most scientifically reliable study was conducted as part of the National Study of Hearing in England (n = 48,313).^[7]^ The results showed an incidence of 10 ± 1% in adults, with 2 ± 8% of respondents describing tinnitus as moderately annoying and 1 ± 6% as severely annoying. The level of serious impact on normal life ability was 0.5%. Findings from studies in Egypt,^[8]^ Japan,^[9]^ and Nigeria^[10]^ suggest that the incidence of tinnitus in these countries is broadly similar to that in Europe and the United States.

The incidence of tinnitus increases with age until 70 years. The results of some studies indicate that it continues to increase thereafter, although others indicate that it is decreasing.^[7]^ The incidence was similar between men and women.^[7]^ The incidence in children is difficult to estimate, but available evidence suggests that the experience of tinnitus in children is common, with figures similar to those in adults.^[11]^ Nonetheless, children are unlikely to be distressed by tinnitus.^[11]^

## 3. Diagnosis of tinnitus

For patients reporting tinnitus symptoms, questions should be asked about the nature of the sound, the duration, the impact on their daily life, and any related symptoms, including hearing difficulties. A history of ear effusion, ear pain, or both suggests possible infectious, inflammatory, or allergic ear disease. Qualitative features of tinnitus described by the patient may also suggest a specific etiology; for example, a growling sound may suggest Meniere disease. Acute tinnitus should be distinguished from persistent tinnitus, although there is no accepted definition for chronic tinnitus. In clinical trials, the definition ranges from 3 to 12 months.^[12,13]^ For patients with tinnitus reporting hearing difficulties and persistent tinnitus, a thorough audiological assessment of the presence, type, severity, and symmetry of hearing loss should be performed.

Standardized questionnaires can be used in both clinical and research settings to assess the severity of tinnitus and its impact on factors in an individual’s daily life.^[14,15]^ These tools can be used for the initial assessment of tinnitus and monitoring of treatment changes. The Tinnitus Handicap Inventory is a widely used assessment tool that is sensitive to changes in treatment severity.^[16]^

## 4. Neuroimaging mechanisms of tinnitus

Tinnitus can be caused by damage to any level of the auditory pathway, but in most cases, it is caused by damage to the cochlea. Thus, acoustic damage in animals has been widely used as a model to study tinnitus, and changes observed at different relays of the auditory pathway have been proposed to represent a neural correlate of tinnitus.^[14,17]^ These studies show that peripheral afferent blocks due to cochlear loss lead to increased spontaneous neuronal activity in the centers of various auditory pathways, all the way down to the auditory cortex. This increase in spontaneous relay activity is reflected in a decrease in neuronal inhibition, with alterations in glycinergic and glutamatergic neurotransmission.^[18]^

An explanation for this increased excitability can be provided by a computational model based on the assumption that the average neuronal firing rate is stabilized by dynamic plasticity.^[19]^ According to the proposed model, central auditory structures respond to a reduction in cochlear input by upregulating neuronal excitability, compensating for it, and thus maintaining a stable average firing rate. This upregulation has also been proposed to amplify “neural noise, “ thus contributing to tinnitus production.^[20]^

Auditory–somatosensory mechanism information integration in the dorsal cochlear nucleus may also be involved in cochlear production. In addition, the integration of auditory and somatosensory information, leading to the modulation of neuronal activity in the central auditory pathway, has also been described to occur in the primary auditory cortex.^[21]^

It has long been suggested that tinnitus pathology involves auditory and nonauditory brain regions.^[22]^ Studies have shown that, although tinnitus perception is associated with abnormal neural activity in the auditory pathway, tinnitus distress is associated with increased coordinated activation of the frontal lobe, limbic system, memory, and autonomic brain areas.^[22]^ In recent years, the central nonauditory area has been considered to be involved not only in tinnitus-related distress but also in the production of auditory hallucinations.^[23]^ The involvement of the central nervous system in tinnitus has been explored using several neuroimaging techniques. In approximately 85% of chronic tinnitus,^[24]^ tinnitus sounds are constantly perceived; resting-state functional measures appear to be well suited to the neuronal correlates of tinnitus. Resting-state networks are based on the spontaneous fluctuations of widely separated brain regions. This is evident in the human brain in the absence of goal-directed neuronal activity and in the absence of external input in the awake resting state.^[25]^ These fluctuations can be measured using fMRI tracking of blood oxygenation level-dependent signals. Such recordings are increasingly being used to understand neurological and psychiatric disorders, many of which have been shown to be associated with alterations in resting-state activity.^[26]^

This is a heterogeneous state in terms of the characteristics of the perceived sound, its varying degrees of associated awareness and distress, its duration, and its commonality.^[27,28]^ As the variability of the clinical signs of tinnitus is expected to be reflected by similar variability in neuron-related structures and functions, it is extremely difficult to identify the underlying neural mechanisms of tinnitus. Among the available cross-sectional studies, 1 study showed a reduction in the volume of the primary auditory cortex^[29]^ in tinnitus patients. Another study showed an increase in auditory cortex volume.^[30]^ Many other studies have not found a variation in this structure in this region.^[31–35]^

Ear disease, especially high-frequency hearing loss, is one of the main risk factors for tinnitus. Therefore, auditory hallucinations are generally considered neuroplastic responses to sensory deprivation.^[14]^ Tinnitus is not simply directly related to an imbalance in firing patterns across the tonal neural array of the damaged cochlea. Sound perception persists even when the input from the ear is eliminated by severing the auditory nerve.^[14]^ Although cochlear abnormalities may be the initial source of tinnitus, subsequent cascades of neural changes in the central auditory system are likely to maintain this condition.

An increase in the spontaneous firing rate of neurons in the central nervous system is a possible cause of tinnitus. Cochlear hearing loss reduces cochlear neural activity, and this reduction in activity in the affected peripheral auditory area downregulates inhibitory cortical processes. This downregulation results in hyperexcitability of central auditory structures.^[20]^ However, it is unclear whether the increase in spontaneous discharge frequency is directly related to the perception of tinnitus. Because tinnitus usually occurs immediately after noise exposure.^[20]^ In animals with normal hearing, neurons respond selectively to characteristic frequencies, and the progression of frequency tuning in the frequency bands of different auditory fields is orderly. Hearing loss results in a disordered pitch topology of the primary auditory cortex, such that neurons with characteristic frequencies within the deprived region adopt the tuning properties of their less affected neighbors at the edge of hearing loss.^[36]^ However, one major psychological finding is inconsistent with the claim that the extension of the tone map at the edge of hearing underpins the perception of tinnitus: the dominant tinnitus tone usually does not fall at the edge of hearing loss.^[37,38]^

The heterogeneity of tinnitus in terms of etiology, neuroimaging, pathophysiology, and clinical features may exacerbate the response of different populations to tinnitus treatment.^[28,39]^ An effective classification system based on the pathophysiology of individual tinnitus symptoms will be key to individualized treatment.^[28]^

## 5. Morbidity mechanism of tinnitus

Currently, the mechanism of tinnitus morbidity remains uncertain. Some scholars believe that in the early stage of tinnitus, the cochlea is the main lesion site, but the central auditory system has an important impact on the pathological progression of tinnitus, in which the occurrence and persistence of tinnitus are mainly regulated by the abnormal activities of the limbic system and cerebral cortex.^[40]^ In other words, the entire auditory conduction pathway is involved in the development and continuation of tinnitus.

Organic lesions in some parts of the peripheral auditory conduction system, muscle spasms, and abnormal blood flow in adjacent tissues may produce abnormal sound sources, thereby causing tinnitus. Some scholars have proposed that tinnitus can be produced in cochlear endings.^[41]^

The central auditory pathway is mainly divided into classical and nonclassical pathways. In the classical pathway, the cochlear nucleus projects to the neurons of the central nucleus of the inferior colliculus and ventral nucleus of the thalamus, and then projects to the primary and secondary auditory cortices. The nonclassical pathway is mainly involved in the regulation of emotional changes in patients with tinnitus and the emotional response of patients to the severity of tinnitus, including the projection of the cochlear nucleus to the dorsal thalamic nucleus and medial thalamic nucleus, and then directly projecting to the secondary auditory cortex and other related cortices such as the amygdala, basal ganglia, and other nonauditory parts.^[42]^ The mechanisms of tinnitus in the central auditory system include changes in neurotransmitters and their receptors, remodeling of the auditory center,^[43]^ and the activity of cochlear nuclei.

It is believed that tinnitus is caused by the joint action and influence of the hearing system, clearing system of tinnitus, and release system of tinnitus. Insufficient auditory input is the inducing factor of tinnitus; that is, after hearing loss, all levels of auditory centers will be activated to produce compensatory responses to tinnitus. At the same time, tinnitus signals will be produced at the level of the thalamus, the most active compensatory site. Only when tinnitus signals are transmitted to the higher auditory cortex can they be perceived. The tinnitus clearing system is in the process of signal transmission. The clearing system for tinnitus is mainly based on the ventromedial frontal cortex. By activating valuable information and eliminating negative information, it mainly plays a “control” role in tinnitus. Therefore, tinnitus is believed to be caused by hearing loss and is regulated by the clearance and release systems during transmission.^[44]^

In addition to the aforementioned factors of auditory system lesions, the possibility of tinnitus caused by systemic diseases should not be ignored. Abnormal sound signals produced by vascular, myogenic, and temporomandibular joint lesions can be transmitted to the brain through the auditory system, leading to perceived or even aggravated tinnitus. Recent studies by Diao Mingfang^[45]^ found that tinnitus was related to sleep quality, and sleep disorders were positively correlated with tinnitus severity.

## 6. Modern medical treatment of tinnitus

Standard treatment is feasible after excluding treatable etiologies associated with tinnitus. In the United Kingdom, the Ministry of Health published a good practice guide for the provision of services for adults with tinnitus,^[46]^ which describes a step-care approach. Primary care physicians provide initial reassurance and diagnosis of simple remediable causes, such as earwax or infection, and referrals to secondary care when tinnitus is severe or accompanied by hearing loss. At this stage, in-depth consultation and appropriate treatment can be performed. Tertiary care was reserved for patients with intractable or severe tinnitus.

In a systematic review, the efficacy of sound therapy methods was inconclusive,^[47]^ but the practice of fitting hearing aids to people with tinnitus associated with hearing loss was self-evident to many clinicians.^[48]^ The use of broadband sound therapy devices is also not supported by the evidence.^[47]^ Relaxation therapy benefit patients.^[49]^ In a systematic review and meta-analysis,^[50,51]^ formal CBT was shown to reduce tinnitus distress, although these studies involved patients who were so distressed that they required referral to a psychiatrist, and the treatment was administered by an expert physician, which may limit its adaptability in general practice.^[52]^ Prescription treatment options for tinnitus are called Tinnitus Retraining Therapy (TRT)^[53]^ and include counseling and generator therapy. A systematic review showed that studies on TRT are of poor quality.^[54]^ A randomized controlled trial (RCT) compared the benefits of consulting elements of CBT and TRT combined with standard care^[55]^ – standard care is an ear, nose, and throat or audiological consultation and an opinion providing a booster or sounder, or both.

No drugs have been approved for the treatment of tinnitus in Europe or North America. Several drugs have been tested to treat tinnitus. Tricyclic antidepressants and selective serotonin reuptake inhibitors are ineffective at reducing tinnitus.^[56]^ However, they may play a role in managing the accompanying psychological distress. A study on benzodiazepine alprazolam^[57]^ reported favorable results, but the quality of the study was not sufficient to draw reliable conclusions. Antispasmodics^[58]^ and drugs for neuropathic pain^[59]^ are generally ineffective, although 1 trial has shown that gabapentin may have a small effect on a subgroup of patients with tinnitus secondary to auditory trauma.^[60]^ Several anticonvulsant drugs, such as aminooxyacetic acid,^[61]^ lamotrigine,^[62]^ and carbamazepine,^[63]^ have been tested for tinnitus treatment. Glutamate is a major excitatory neurotransmitter of the auditory system. Therefore, various antagonist drugs such as memantine, flupirtine, and neramexane have been investigated for this purpose. However, none have shown a benefit for tinnitus.^[64–66]^

## 7. Cognition of tinnitus in traditional Chinese medicine (TCM)

As early as the era of Huangdi Neijing, there are relevant records of tinnitus and deafness. It is recorded in Suwen Maijie that “the so-called tinnitus is caused by the rise of Yang Qi in all things,”^[67]^ which is defined as tinnitus. The increase in Yang Qi is a mechanism of tinnitus. It is said in Suwen Jue Lun that “the syncope of Shaoyang leads to sudden deafness” and “the syncope of hand Taiyang leads to deafness.” Deafness is caused by the adverse flow of meridian qi, which is consistent with the jumping of yang qi. The above is deafness caused by excess syndrome. It is recorded in the Miraculous Pivot: Oral Questions: “What is the cause of the ringing in the ears??” Qi Bo said, ‘The ear is the place where the meridians gather. Therefore, when the stomach is empty, the meridians are abundant, and when the stomach is empty, the meridians slip downward. The ears ringed when the meridians were exhausted. Therefore, the upper qi is insufficient, the brain is dissatisfied with it, and the ears are suffering from it.^[68]^

## 8. Etiology and pathogenesis of tinnitus

1. Xue Boshou, a Chinese medicine expert, believes that the 5 Zang-organs and 6 Fu organs are closely related to tinnitus, so the etiology and pathogenesis of tinnitus are discussed from the 5 Zang-organs.^[69]^ For example, from the kidney theory, “Miraculous Pivot · Pulse Degree” says: “Kidney Qi passes through the ear, and when the kidney is in harmony, the ear can hear the 5 tones.” The kidneys were open in the ear. If the kidney essence is deficient, the sea of the marrow loses its fullness. If the sea of the marrow is insufficient, the brain will develop tinnitus. Just as it is said in the Miraculous Pivot Sea Treatise: “If the sea of marrow is insufficient, the brain will turn to tinnitus.” “Miraculous Pivot · Jue Qi” also says: “The brain and marrow disappear, the shin is sour, and the ears are ringing..” The heart was placed in the ear for those who followed it. If the heart is deficient, the ear loses its nourishment, which leads to tinnitus. For example, The Complete Collection of Ancient and Modern Medicine: The Gate of Ear Diseases: “Worry and worry will hurt the heart, and the consumption of heart blood will lead to deafness.” In fact, heart fire disturbs clear orifices, which can also lead to tinnitus. For example, it is said in The True Story of Medicine: Ear Diseases: “When the heart fire is. From the viewpoint of the liver, depression and yang hyperactivity can attack the ears and eyes and cause tinnitus. For example, Su Wen Liu yuan Zheng Ji Da Lun also says: “The hair of wood depression is even tinnitus and dizziness.” Su Wen Zhi Zhen Yao Da Lun also says: “The victory of Jueyin is tinnitus and dizziness..” In the spleen and stomach, this deficiency can cause tinnitus. For example, it is said in the Miraculous Pivot: Oral Questions: “The ear is the place where the meridians gather, so the stomach is empty and the meridians are weak. When the meridians are weak, they slip downward, and the meridians are exhausted, so tinnitus occurs.” In fact, phlegm-heat tinnitus occurs. For example, it is said in the Introduction to Medicine: Ear: “The phlegm fire rises because of the heat in the. According to lung theory, the knot points of the lung meridian are in the ear, which are in charge of hearing. It is said in Volume 23 of the Origin of Miscellaneous Diseases: “The lung governs qi, and the qi of the whole body runs through the ear, so it can be used for hearing.” Therefore, deficiency or disadvantage of lung qi can lead to tinnitus and deafness. In addition, zang-fu organs interact with each other, the pathogenesis is transmitted and changed, and the restriction is unbalanced. One zang-fu organ is the beginning, and other zang-fu organs can be involved, such as deficiency of kidney water or hyperactivity of heart fire, resulting in disharmony between the heart and the kidney and tinnitus. Volume III of Records of Differentiation of Syndromes says: “When the heart and the kidney intersect, the upper and lower parts of the body can be clear and calm, so that they can see and hear. If the kidney does not communicate with the heart. Therefore, hyperactivity of the 5 Zang organs is harmful to the system, and harmony leads to clear orifices and benefits, whereas disharmony leads to tinnitus.
2. When the wind is moving, the ears are ringing, clear orifices are in the upper part, and the wind is attacking the upper part by the Yang evil. Li Gao once said: “On the top of the mountain, only the wind can reach it.” Therefore, invasion of the external wind or disturbance of the internal wind can disturb the clear orifices, resulting in tinnitus. Tinnitus can be caused by the invasion of external wind which disturbs the clear orifices. For example, it is pointed out in the Treatise on the Causes and Symptoms of Various Diseases that “when the menstrual blood is deficient due to labor, the blood and qi are insufficient, and the meridians are deficient. The wind evil takes advantage of the deficiency and enters the ear along with the meridians, striking each other with qi.” It is also stated in the Sheng Ji Zong Lu, Volume 114: “When the kidney qi is deficient, the wind evil dries up, and the heart is overloaded with thinking. If the Zang–Fu organs are in harmony with each other, the internal and external organs are in harmony, and the upper and lower organs communicate with each other, then “Yin and Yang are balanced, and the spirit is treated.” If the zang-fu organs are out of harmony, the restriction is unbalanced, and an internal wind can also be generated. Excess syndrome is characterized by liver depression transforming into fire, excessive heat generating wind, and wind stirring, which is characterized by water not containing wood, internal disturbance of deficient wind, and wind stirring due to yin deficiency. “The wind passes through the liver,” the liver fails to disperse the qi, and the qi movement fails to regulate. “The excess of qi is fire,” the qi depression transforms into fire and generates wind, and the wind and fire interfere with each other, thus disturbing the ear orifices, that is, the occurrence of wood depression is tinnitus. Alternatively, the liver and kidney essence is deficient, the marrow sea is not filled, the clear orifices are not nourished, the kidney water is deficient, the water does not contain wood, the wind due to yin deficiency moves, and the wind and Yang are disturbed, resulting in tinnitus-like cicadas.

Thus it can be seen that the pathogenesis of tinnitus involves the 5 Zang-organs, and all the 5 Zang-organs can cause tinnitus, not the only 1. If the Zang–Fu organs are out of harmony, the external wind can easily attack the Yang position; the Zang–Fu organs generate wind, and the wind can easily cause singing. The basic pathogenesis of tinnitus is disharmony of the 5 viscera, wind is the main pathological factor, and clinical signs are characterized by deficiency and excess, so the treatment should be based on harmony of the 5 viscera and holistic syndrome differentiation and treatment.

## 9. Research progress on syndrome types of tinnitus patients

As we all know, the basic principle of TCM in the treatment of diseases is syndrome differentiation and treatment. Many researchers have studied the syndrome types of tinnitus. Wei Wei^[70]^ classified 166 cases of tinnitus according to syndrome differentiation. The results showed that wind-heat disturbance type and kidney essence deficiency type were the most common, but liver-fire, phlegm-fire, qi stagnation, and blood stasis also accounted for a considerable proportion. Cong Pin et al^[71]^ classified 134 cases of tinnitus according to their syndrome differentiation. The results showed that invasion of pathogenic wind and deficiency of the liver and kidney were the most common. Feng Juan et al^[72]^ investigated 152 patients with tinnitus and found that deficiency of Qi and blood was the most common type, followed by deficiency of kidney essence.

## 10. TCM for tinnitus

Li Shufang et al^[73]^ designed an RCT to explore the effect of modified Sitenglongmu Decoction combined with mecobalamin in the treatment of nervous tinnitus. In this study, 220 patients with tinnitus were randomly divided into 2 groups. One hundred ten cases in the control group were treated with mecobalamin, and 110 patients in the observation group were treated with mecobalamin and Siteng Longmu Decoction. One week was the course of treatment in both groups, and the curative effect was evaluated after 4 courses of treatment. The severity of tinnitus was evaluated before treatment and after 2 and 4 courses of treatment, and the peak value of basal artery blood flow at the end of diastole, peak value of basal artery blood flow at the end of systole, and the content of central neurotransmitters were detected. The safety of the 2 groups was evaluated during treatment. Conclusion The treatment of SITENGLONGMU decoction combined with mecobalamin has a significant effect on neurogenic tinnitus, which can effectively reduce the degree of tinnitus. Its mechanism may be related to the regulation of 5-hydroxytryptamine (5-HT) and γ-aminobutyric acid (GABA) levels, and it is helpful to improve local microcirculation with high safety.

Liang Zimeng et al^[74]^ designed an RCT to observe the clinical efficacy of a modified Longdan Xiegan Decoction in the treatment of tinnitus with dampened heat in the liver and gallbladder. In this study, 80 patients with mild tinnitus of the liver-gallbladder damp-heat type were randomly divided into 2 groups. Forty patients (50 ears) in the control group were treated with conventional Western medicine, and 40 patients (50 ears) in the treatment group were treated with a modified Longdan Xiegan Decoction on the basis of the control group, and both groups were treated for 4 weeks. The degree of tinnitus, Hamilton Depression Scale score, and serum platelet activating factor level were compared between the 2 groups before and after treatment, and the onset time and clinical efficacy were compared between the 2 groups. Conclusion: Longdan Xiegan Decoction is effective in the treatment of tinnitus with damp-heat in the liver and gallbladder, which can effectively improve the influence of tinnitus on mood and reduce the level of serum platelet activating factor.

Zhang Yijun^[75]^ observed the clinical effect of Xiaochaihu Decoction in tinnitus treatment. In this study, 72 patients with tinnitus were divided into a TCM group and a Western medicine group according to a random number table, with 36 patients in each group. The Western medicine group was treated with mecobalamin capsules, while the TCM group was treated with Xiaochaihu Decoction, and the western medicine group.The curative effects, TCM syndrome scores (dizziness, soreness of waist and knees, tinnitus, and deafness), hearing thresholds of the left and right ears, and tinnitus severity scores before and after treatment were compared between the 2 groups. Conclusion: The treatment of tinnitus with Xiaochaihu Decoction can improve the clinical curative effect, improve the clinical signs and symptoms, reduce the degree of tinnitus, and improve hearing.

## 11. Acupuncture treatment of tinnitus

Li Chenglong^[76]^ designed an RCT to observe the clinical efficacy of “Tongdu Tiaoshen” acupuncture in the treatment of subjective tinnitus and to explore its possible mechanism. In this study, 92 patients with subjective tinnitus were randomly divided into an acupuncture group (46 cases, 5 cases of loss) and a drug group (46 cases, 2 cases of loss). The acupuncture group was treated with acupuncture at Shuigou, Yintang, Shenting, Baihui, Fengfu, Dazhui, Zhongzhu, Tinghui, and Yifeng on the affected side for 30 minutes each time, once every other day; the drug group was orally administered ginkgo leaf tablets (40 mg each time) and mecobalamin tablets (0. 5 mg each time) 3 times daily. Both groups were treated for 4 weeks. The tinnitus severity score, visual analog scale (VAS) score, and self-rating emotional scale scores were observed before and after treatment in the 2 groups. The serum brain-derived neurotrophic factor content was measured before and after treatment, and the clinical efficacy of the 2 groups was evaluated. This study found that “Tongdu Tiaoshen” acupuncture can improve the severity of tinnitus, tinnitus loudness, and poor mood in patients with subjective tinnitus, and its mechanism may be related to regulating the content of serum brain-derived neurotrophic factor and affecting the plasticity of the auditory center.

Tang Zhigang et al^[77]^ designed an RCT to observe the clinical efficacy of acupuncture in the treatment of tinnitus of the kidney essence deficiency type. In this study, 60 patients with tinnitus of the kidney essence deficiency type were randomly divided into a treatment group (30 cases) and a control group (30 cases). The control group was treated with conventional acupuncture, while the treatment group was treated with acupoint selection and acupuncture according to the Dunhuang Moxibustion Classic Picture. The severity of tinnitus was assessed before and after treatment, and the scores of Tinnitus Impairment Scale (tinnitus handicap inventory, THI) and VAS were compared between the 2 groups before and after treatment. Clinical efficacy was compared between the 2 groups. The study found that the clinical efficacy of acupuncture based on the Dunhuang Moxibustion Classic Picture is better than that of conventional acupuncture in treating nervous tinnitus of the kidney essence deficiency type.

Liu Juan et al^[78]^ designed an RCT to observe the clinical efficacy of acupuncture at the points of host and guest origin and collaterals combined with conventional acupuncture in the treatment of tinnitus of the kidney essence deficiency type and the effects on serum levels of soluble vascular cell adhesion molecule-1 (sVCAM-1) and intercellular adhesion molecule-1 (ICAM-1). In this study, 98 patients with tinnitus of the kidney essence deficiency type were randomly divided into study and control groups, with 49 patients in each group. The control group received conventional acupuncture treatment, and the study group was additionally treated by matching acupoints of host and guest origin and collaterals. TCM syndrome integral, pure tone audiometry of different frequencies, average hearing threshold of air conduction, tinnitus disability assessment scale (THI), and serum oxidative stress indicators (total antioxidant capacity, glutathione peroxidase, and superoxide dismutase) were observed before and after treatment. Levels of sVCAM-1 and ICAM-1 were measured and compared between the 2 groups. Conclusion: The combination of acupuncture at acupoints of host and guest origin and collaterals and routine acupuncture can reduce the symptoms of tinnitus, downregulate the levels of sVCAM-1 and ICAM-1 in serum, relieve oxidative stress, and improve hearing.

Zhang Liwen et al^[79]^ designed an RCT to observe the clinical efficacy of Linggui Bafa acupuncture in the treatment of sudden deafness and its effects on hearing levels and hemorheological indicators. In this study, 103 patients with sudden deafness were randomly divided into a control group (n = 51) and an observation group (n = 52). Both groups were treated with conventional Western medicine, the control group was treated with ordinary acupuncture, and the observation group was treated with Linggui Bafa acupuncture. The clinical efficacy, time of symptom improvement, hearing level, and changes in hemorheology indices before and after treatment were observed, and the adverse reactions of the 2 groups were compared. Conclusion: Linggui Bafa acupuncture can improve the symptoms of tinnitus and vertigo in patients with sudden deafness, regulate the level of hearing, regulate the level of hemorheology, and reduce the occurrence of adverse reactions, with good curative effect.

## 12. Treatment of tinnitus by acupuncture combined with medicine

Fan Hongxia et al^[80]^ designed an RCT to study the clinical efficacy of a temporal three-needle combined with breathing method in the treatment of nervous tinnitus of the liver-qi stagnation type. In this study, 104 patients with tinnitus in the nervous system were randomly divided into a treatment group (n = 52) and a control group (n = 52). The treatment group was treated with a temporal three-needle breathing method, whereas the control group was treated with conventional oral medicine. The number of cases and changes in tinnitus disability assessment scale (THI) scores were observed before and after treatment in both groups. Studies have found that the temporal three-needle combined with breathing method has a significant effect on tinnitus in the nervous system, which can effectively alleviate tinnitus and its accompanying symptoms and improve the degree of tinnitus disability.

Yan Ajian^[81]^ designed an RCT to observe the efficacy of Erlong Zuoci Pill combined with sound therapy in the treatment of chronic tinnitus with kidney essence deficiency. In this study, 80 patients with chronic tinnitus due to kidney essence deficiency were randomly divided into an observation group and a control group, with 40 patients in each group. The control group was treated with sound therapy, while the observation group was treated with deafness Zuoci pill on the basis of the control group. The course of treatment was 3 months in both groups. The clinical efficacy and incidence of adverse reactions were compared between the 2 groups, and changes in the tinnitus disability assessment scale (THI) score, tinnitus severity score, Pittsburgh Sleep Quality Index (PSQI) score, and TCM syndrome score were compared between the 2 groups before and after treatment. Conclusion: Sound therapy combined with Erlong Zuoci Pill can improve the clinical efficacy, clinical signs and symptoms, and sleep quality of patients with chronic tinnitus of the kidney essence deficiency type.

Wang Xin^[82]^ designed an RCT to observe the clinical efficacy of acupuncture combined with medicine in the treatment of patients with nervous tinnitus of the liver-qi stagnation type and the effect on the expression level of serum central neurotransmitters. In this study, 94 patients with nervous tinnitus of the liver-qi stagnation type were randomly divided into a treatment group (n = 47) and a control group (n = 47). The control group was treated with Flunarizine Hydrochloride Capsules, Ginkgo Leaf Dropping Pills and Mecobalamin Tablets, while the observation group was treated with Xiaoyao Powder combined with acupuncture on the basis of the control group. Both groups were treated for 20 days. The clinical efficacy was compared between the 2 groups. TCM syndrome scores, Tinnitus Evaluation Scale scores, pure tone hearing threshold levels, and serum central neurotransmitter (GABA, growth associated protein 43, and 5-HT) expression levels were observed before and after treatment. Conclusion: Acupuncture combined with TCM has a significant clinical effect on tinnitus of the liver-qi stagnation type, which can effectively improve the symptoms of tinnitus and improve the hearing level, which is related to the regulation of the release of central neurotransmitters such as GABA, growth associated protein 43, and 5-HT.

Su Meiyi et al^[83]^ designed an RCT to observe the clinical efficacy of acupuncture combined with long chiropractic therapy in the treatment of cervicogenic tinnitus. In this study, 60 patients with cervicogenic tinnitus were randomly divided into the treatment and control groups, with 30 patients in each group. The treatment group was treated with acupuncture combined with long chiropractic therapy, whereas the control group was treated with acupuncture combined with traditional massage therapy. Seven days for the course of treatment, a total of 2 courses of treatment were administered. After 2 weeks of treatment, the clinical efficacy of the 2 groups was evaluated, and changes in VAS and THI scores before and after treatment were observed. Changes in blood flow velocity of the vertebral artery and basilar artery were compared between the 2 groups before and after treatment. The recurrence rates were compared between the 2 groups. Studies have found that acupuncture combined with Long chiropractic therapy for cervicogenic tinnitus can significantly improve the clinical signs and symptoms of patients, improve blood circulation, thereby improving the quality of life of patients, and has a significant effect.

## 13. Brief summary

The mechanism of tinnitus morbidity is complex, and there are many related factors. The clinical treatment of tinnitus in modern Chinese medicine is multi-level and multi-faceted, TCM treatment is not to treat the kidney alone, but to start with the 5 internal organs and physical conditioning, while paying attention to the psychological and emotional intervention of patients, controlling the susceptible factors, combining with the characteristics of tinnitus attack, accompanying symptoms and tongue and pulse, the treatment is based on disease differentiation, syndrome differentiation and body differentiation. Its treatment methods are various, including TCM, acupuncture, pricking blood, acupoint injection, Daoyin, emotional therapy and control of susceptible factors, which have the characteristics of holistic concept and syndrome differentiation and treatment. Compared with Western medicine neurotrophic therapy, the effect of syndrome differentiation and treatment combined with holistic conditioning therapy in TCM is more significant. In order to regulate the functions of the viscera and meridians, smooth the qi movement of the whole body, make its ascending and descending, and smooth the flow of qi in an orderly manner to alleviate tinnitus. Although TCM treatment of tinnitus has achieved satisfactory results, there are still the following problems: (1) tinnitus is a subjective symptom, the clinical etiology and pathogenesis of tinnitus are not clear at this stage, and there is no uniform standard for the evaluation of curative effect, so the clinical curative effect may be affected by the subjective factors of patients. (2) Some clinical case reports or clinical studies have some shortcomings, such as simple research methods, nonstandard methods, small sample size, low degree of demonstration, and lack of multi-level, high-quality, large-data clinical control studies, which require more standardized research methods to improve the quality of research. (3) There may be regional differences in the treatment of the TCM constitution, so regional cooperation should be strengthened in the future, and large sample, multi-level, and high-quality clinical research should be carried out to provide evidence-based medical evidence for the treatment of tinnitus with TCM. (4) Although TCM has advantages in the treatment of tinnitus, the relationship between tinnitus and viscera, qi, blood, yin, and yang, and the mechanism of morbidity have not yet formed a definite theoretical system.

## Author contributions

**Conceptualization:** Wenhui Hu.

**Data curation:** Wenhui Hu, Dongye Xu, Qingchang Xing.

**Formal analysis:** Wenhui Hu.

**Funding acquisition:** Wenhui Hu.

**Investigation:** Wenhui Hu.

**Methodology:** Wenhui Hu.

**Project administration:** Wenhui Hu.
